# Comparative Study on International Research Hotspots and National-Level Policy Keywords of Dynamic Disaster Monitoring and Early Warning in China (2000–2021)

**DOI:** 10.3390/ijerph192215107

**Published:** 2022-11-16

**Authors:** Jie Gao, Wu Zhang, Chunbaixue Yang, Rui Wang, Shuai Shao, Jiawei Li, Limiao Zhang, Zhijian Li, Shu Liu, Wentao Si

**Affiliations:** 1 School of Innovation and Entrepreneurship, Shandong University, Qingdao 266237, China; 2 School of Finance, Hunan University of Finance and Economics, Changsha 410025, China; 3 Institute of Marine Science and Technology, Shandong University, Qingdao 266237, China; 4 Yellow Sea Fisheries Research Institute, Chinese Academy of Fishery Sciences, Qingdao 266237, China; 5 Information Materials and Intelligent Sensing Laboratory of Anhui Province, Anhui University, Hefei 230601, China; 6 School of Environment, Tsinghua University, Beijing 100084, China; 7 Institute for Intelligent Society Governance, Tsinghua University, Beijing 100084, China; 8 School of Public Administration, Shandong Normal University, Jinan 250014, China

**Keywords:** dynamic disaster monitoring and early warning, research hotspot, policy keyword, policy document quantification, knowledge map visualization, emergency management, disaster prevention and mitigation

## Abstract

For more than 20 years, disaster dynamic monitoring and early warning have achieved orderly and sustainable development in China, forming a systematic academic research system and top-down policy design, which are inseparable from the research of China’s scientific community and the promotion of government departments. In the past, most of the research on dynamic disaster monitoring and early warning focused on specific research in a certain field, scene, and discipline, while a few studies focused on research review or policy analysis, and few studies combined macro and meso research reviews in academia with national policy analysis for comparative analysis. It is necessary and urgent to explore the interaction between scholars’ research and policy deployment, which can bring theoretical contributions and policy references to the top-down design, implementation promotion, and academic research of China’s dynamic disaster monitoring and early warning. Based on 608 international research articles on dynamic disaster monitoring and early warning published by Chinese scholars from 2000–2021 and 187 national policy documents published during this period, this paper conducts a comparative analysis between the knowledge maps of international research hotspots and the co-occurrence maps of policy keywords on dynamic disaster monitoring and early warning. The research shows that in the stage of initial development (2000–2007), international research articles are few and focused, and research hotspots are somewhat alienated from policy keywords. In the stage of rising development (2008–2015), after the Wenchuan earthquake, research hotspots are closely related to policy keywords, mainly in the fields of geology, engineering disasters, meteorological disasters, natural disasters, etc. Meanwhile, research hotspots also focus on cutting-edge technologies and theories, while national-level policy keywords focus more on overall governance and macro promotion, but the two are gradually closely integrated. In the stage of rapid development (2016–2021), with the continuous attention and policy promotion of the national government, the establishment of the Ministry of Emergency Management, and the gradual establishment and improvement of the disaster early warning and monitoring system, research hotspots and policy keywords are integrated and overlapped with each other, realizing the organic linkage and mutual promotion between academic research and political deployment. The motivation, innovation, integration, and transformation of dynamic disaster monitoring and early warning are promoted by both policy and academic research. The institutions that issue policies at the national level include the State Council and relevant departments, the Ministry of Emergency Management, the Ministry of Water Resources, and other national ministries and commissions. The leading affiliated institutions of scholars’ international research include China University of Mining and Technology, Chinese Academy of Sciences, Wuhan University, Shandong University of Science and Technology, and other institutions. The disciplines involved are mainly multidisciplinary geosciences, environmental sciences, electrical and electronic engineering, remote sensing, etc. It is worth noting that in the past two to three years, research and policies focusing on COVID-19, public health, epidemic prevention, environmental governance, and emergency management have gradually increased.

## 1. Introduction

China is one of the countries with the most serious natural disasters in the world [1]. With global climate change and the rapid economic development and urbanization in China, the pressure on resources, environment, and ecology in China has intensified, and the situation of natural disaster prevention and response has become more severe and complicated [1,2,3]. The Chinese government adheres to the people-oriented principle, always puts the protection of public life and property safety first, and integrates disaster monitoring and early warning, disaster prevention, mitigation, and relief into economic and social development planning as an essential guarantee for achieving sustainable development [1,2,3,4,5].

In recent years, China has actively promoted the modernization of natural disaster prevention and control systems and capacity, deepened the reform of emergency management systems and mechanisms with China’s characteristics, established the Emergency Management Ministry, basically established the natural disaster management system with overall coordination and division of responsibilities, continuously optimized the natural disaster management system, and further enhanced the natural disaster prevention and control capacity by organizing and implementing critical projects of natural disaster prevention and control [1,2,3,4,5]. China is also strengthening the comprehensive management of the whole process of disaster types and the optimal management of emergency force resources. Disaster information is submitted more timely, multi-department and cross-regional coordination (such as comprehensive monitoring and early warning, major risk judgment, material allocation, emergency rescue, etc.) is more efficient, and the disaster relief capability is significantly improved [2,3,4,5]. Meanwhile, China has actively practiced the Community of Shared Future for Mankind concept, and made remarkable progress in implementing the United Nations 2030 Agenda for Sustainable Development and Sendai Framework for Disaster Risk Reduction from 2015 to 2030 [2,5,6,7]. The cooperation under the regional cooperation frameworks such as the Shanghai Cooperation Organization and China-ASEAN has become more pragmatic, and the exchanges and cooperation with the countries that jointly built the “belt and road initiative” have been expanding. Moreover, the China International Rescue Team and China Rescue Team have actively participated in international rescue operations, which fully demonstrates the image of a responsible big country, and the achievements of international cooperation and exchanges are remarkable [2,5,6,7].

For more than 20 years, many scholars around the world have carried out continuous and in-depth research in dynamic disaster monitoring and early warning, disaster reduction, disaster prevention and relief, disaster management and emergency management, and other related fields [8,9,10,11,12,13,14,15,16,17,18,19]. Some scholars have carried out a series of studies and exploration on the selection and application of methods based on water and soil environment management and disaster monitoring and early warning, and in terms of recently occurring pandemics (COVID-19) and recently used feature selection methodologies (such as machine learning models, data mining techniques, artificial intelligent models, big data analysis, and other integrated methodologies) [14,20,21,22,23]. The quantity and quality of Chinese scholars’ papers have been increased and strengthened after entering the new century, and the close relationship with national policies has also been strengthened, forming a good relationship of mutual promotion [24,25,26,27,28,29,30,31,32,33]. The research of Chinese scholars in relevant fields includes not only the research on prevention and early warning of natural disasters, postdisaster assessment, and treatment in a certain field [34,35,36,37,38,39,40], but also the research on the construction of the entire disaster early warning monitoring system, disaster management, and emergency management system [3,4,5,6,41,42,43]. Chinese scholars have also continued to conduct cooperation and exchanges at home and abroad. They have not only discussed disaster monitoring and early warning, disaster reduction, prevention and relief, environmental governance, emergency management, and other aspects in the international field [44,45,46], but also studied and explored disaster monitoring and early warning, prevention and assessment of various disasters and emergency events, governance system and governance capacity development, institutional mechanism construction, policy analysis, and other aspects in China [47,48,49].

In recent years, Chinese scholars have begun to increase their research on the construction of dynamic disaster monitoring and early warning systems, major projects, emergency management systems and mechanisms, algorithms and methods, including technical analysis focusing on a certain field, as well as a relatively macro overview and research on system policies [41,50,51,52,53,54,55,56,57]. Moreover, the research on the COVID-19 epidemic, emergencies, public health, environmental governance, and emergency response has gradually crossed with the research in the field of dynamic disaster monitoring and early warning [20,42,46,58]. In general, China’s national policies and academic research in the field of dynamic disaster monitoring and early warning have been gradually and orderly promoted and improved according to the five-year plan and the deployment of the medium- and long-term plan. The relevant research of Chinese scholars is gradually increasing, and the international influence is also growing. Many scholars have conducted many specific academic or policy studies in a certain field, scene, or discipline [3,4,5,6,54,55,56,57], but few studies combine policy analysis and research with academic research hotspots in related fields, carry out comparative analysis, and discuss the evolution and interaction between academic communities and government departments and other organizations. Some scholars have carried out bibliometric analysis of academic research in the field of dynamic disaster monitoring and early warning [3], and carried out a summary of a specific field [45], while others have carried out comparative studies in other fields and policies [59]. At present, there are few studies that compare international research hotspots of Chinese scholars with national policy themes.

The research design and novelty of this paper lie in a simple comparative analysis of policy themes and academic research hotspots. Few scholars have carried out such comparative analysis in the field of dynamic disaster monitoring and early warning before, combining the visual analysis of a scientific metrological knowledge map with the quantitative analysis of policy documents emerging in recent years, and conducting research on the analysis and interaction between the research level and the policy level, This paper discusses the fluctuation, evolution, and mutual influence of academic research and policy practice in different periods. By analyzing the development of China’s dynamic disaster monitoring and early warning policy and the evolution of the theme, and conducting evolutionary analysis and comparative research with international research hotspots and themes of Chinese scholars, this study can gain insight into the relevant policy layout and top-level design of China’s dynamic disaster monitoring and early warning, and provide some academic references and policy suggestions for the modernization, internationalization, innovation, transformation, and sustainable development of academic research.

## 2. Knowledge Map Visualization Analysis of International Research Evolution of Dynamic Disaster Monitoring and Early Warning in China

A knowledge map is an image and method that can express the relationship between the development and structure of scientific knowledge in bibliometrics, scientometrics, and knowledge metrology, which could be used to carry out evolutionary analysis and structural analysis on research hotspots and keywords, and provide scientific visual analysis and a decision-making basis for knowledge accumulation, hot frontiers, development trends, and policy analysis in a certain field [60,61,62,63].

Through the visualization analysis of the keywords and knowledge network structures of Chinese scholars’ international cooperation research articles in the field of dynamic disaster monitoring and early warning, this study further explores and reveals the research interests, research hotspots, and research directions of Chinese scholars in this field, so as to further carry out linkage analysis with policy themes, and reveal the interactive relationships and mutual influences between Chinese scholars’ international research and national policy layout and promotion in the field of dynamic disaster monitoring and early warning.

This research selected the international representative authoritative database, the “Web of Science Core Collection” of Clarificate Analytics’s Web of Science database (http://www.isiknowledge.com/, accessed on 10 October 2022) to obtain Chinese scholars’ international cooperative research articles. We retrieved the related articles on relevant topics such as “dynamic disaster monitoring and early warning”, “dynamic disaster monitoring”, and “dynamic disaster monitoring early warning”, and set the period from 2000 to 2021, and “China” was selected and refined for “Country/Region”. Through continuous refining and screening, we have obtained 608 articles related to the international cooperative research of Chinese scholars in the field of dynamic disaster monitoring and early warning over the past 20 years from 2000 to 2021. These 608 selected articles are the basic data for our various data analyses and knowledge map visualization analysis.

### 2.1. The Stage Distribution and Highly Cited Articles of China’s International Research on Dynamic Disaster Monitoring and Early Warning

**Figure 1:**
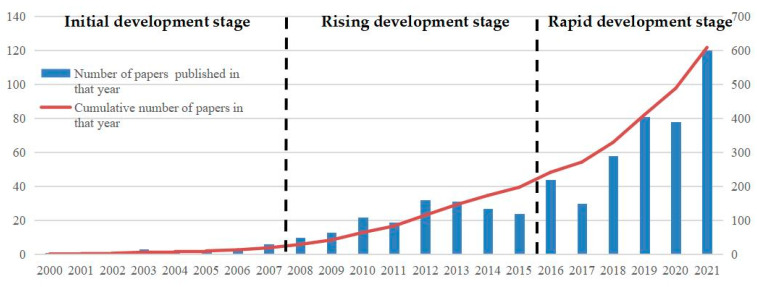
The annual number and stage distribution of China’s dynamic disaster monitoring and early warning international research (2000–2021).

By analyzing the international cooperation research carried out by relevant scholars around the world, especially the evolution rules and trends of papers published in high-level conferences and essential journals, scholars could gain insight into the evolution, knowledge accumulation, and development direction of hot and frontier areas in a certain field [60,61,62,63]. After sorting out and analyzing the relevant papers of Chinese scholars’ international cooperation research in the field of dynamic disaster monitoring and early warning, we have drawn the trend chart of annual article number and stage distribution of China’s dynamic disaster monitoring and early warning international research from 2000 to 2021, as shown in Figure 1.

Figure 1 shows that although the relevant papers published by the international research on dynamic disaster monitoring and early warning in our country fluctuate in some years or stages, the overall trend is gradually increasing, and the growth rate is faster in the later period. According to the cumulative number of papers, growth rate, policy changes, and turning points, we divide the growth of papers into three stages: (1) The first stage is the initial development stage (2000–2007), this stage is the initial stage, it shows a slow trend of year-to-year growth. (2) The second stage is the rising development stage (2008–2015), the number of papers in this stage shows a fluctuating growth trend. Among them, more papers were published from 2012 to 2013, but the overall average annual number of papers published is still no more than 40. (3) The third stage is the rapid development stage (2016–2021). The number of papers published in this stage shows a trend of rapid growth. Among them, the average annual number of publications from 2016 to 2018 is about 40, while in the past three years (2019–2021), the number of papers published has increased rapidly, and the average number of papers published per year has exceeded 80. Although there are slight fluctuations, the number and speed of the growth of papers indicate that the related research in this field has entered a new development period.

After further searching and refining the analysis of these 608 papers in the core collection of the Web of Science database, we can obtain six highly cited papers of international research on dynamic disaster monitoring and early warning in China selected from the database. Specifically, “highly cited paper” means this highly cited paper received enough citations to place it in the top 1% of relevant academic fields based on a highly cited threshold for the field and publication year. The specific information and citations of these six papers are shown in Table 1.

Table 1 shows that these six highly cited papers include both review articles and research articles; there is both external collaborative research between Chinese scholars and foreign scholars and internal cooperation between domestic scholars. Among them, there is also a “hot paper” identified in the Web of Science database, which is the article “Research on theory, simulation, and measurement of stress behavior under the cooperation regenerated roof condition” written by Li Xuelong, Chen Shaojie, and other scholars [55], ranked 5th in Table 1. The mark “hot paper” means this hot paper was published in the past two years and received enough citations in May/June 2022 to place it in the top 0.1% of papers in the academic field of engineering. Overall, these six highly cited papers are all from 2020 to 2021, which means that in the third stage (2018–2021), the rapid development stage of the publication of papers in related fields, there is not only rapid growth of the number and speed of papers, but also the overall improvement in quality. The relevant international research papers of Chinese scholars in this field are increasingly cited and valued by the academic community.

### 2.2. The Main Discipline Categories, Countries, and Regions of China’s International Research on Dynamic Disaster Monitoring and Early Warning

Table 2 shows the refinement and statistical analysis of the disciplinary categories of international research on dynamic disaster monitoring and early warning by Chinese scholars. As the paper retrieval and statistical analysis are carried out in the Web of Science (WOS) database, here we use WOS discipline categories as the standard of discipline classification. As can be seen from Table 2, the top 10 disciplines are roughly Geosciences Multidisciplinary (117 articles), Environmental Sciences (105 articles), Engineering Electrical Electronic (83 articles), and Remote Sensing (82 articles), Water Resources (51 articles), Computer Science Information Systems (49 articles), Engineering Civil (47 articles), Imaging Science Photographic Technology (46 articles), Engineering Geological (43 articles), and Meteorology Atmospheric Sciences (39 articles) according to the number of papers.

In addition, the international research of Chinese scholars on dynamic disaster monitoring and warning also involves telecommunications, mechanics, mining mineral processing, environmental engineering, and multidisciplinary engineering, and other multidisciplinary and interdisciplinary sciences categories.

Table 3 shows the results of refinement and statistical analysis of the leading countries and regions cooperating with Chinese scholars on dynamic disaster monitoring and early warning. The table shows that except for China, the leading cooperation countries and regions are the USA (39 articles), Australia (10 articles), Japan (10 articles), Pakistan (9 articles), England (8 articles), Germany (6 articles), Canada (5 articles), Spain (4 articles), Austria (4 articles), Belgium (4 articles).

In addition, the countries and regions where the scholars and institutions cooperating with Chinese scholars on dynamic disaster monitoring and warning are located are Finland, France, Italy, Singapore, Scotland, Netherlands, New Zealand, Switzerland, etc.

### 2.3. The Main Institutions and Scholars of China’s International Research on Dynamic Disaster Monitoring and Early Warning

Table 4 shows the refinement and statistical analysis of the main affiliations of international research on dynamic disaster monitoring and early warning by Chinese scholars. From Table 4, according to the number of published papers, the top 10 major institutions are China University of Mining Technology (95 articles), Chinese Academy of Science (87 articles), University of Chinese Academy of Science CAS (30 articles), Wuhan University (28 articles), Shandong University of Science and Technology (26 articles), China University of Geosciences (24 articles), Chongqing University (22 articles), Helmholtz Association (18 articles), University of Science Technology Beijing (17 articles), Xi’an University of Science and Technology (17 articles). Among them, China University of Mining and Technology and the Chinese Academy of Sciences belong to the first group in terms of the number of papers published in this field. This is inseparable from the traditional advantageous disciplines and historical accumulation of China University of Mining and Technology. Various scientific research institutes and institutions under the Chinese Academy of Sciences system also have advantages. At the same time, in addition to relevant domestic institutions, there are also institutions in other countries and regions participating in cooperative research. For example, the Helmholtz Association in Germany has Chinese scholars engaged in international cooperation and exchanges, such as cooperative research and visiting scholars’ work.

In addition, many Chinese scholars engaged in research on dynamic disaster monitoring and warning and published international articles, as well as many relevant universities and research institutions, government departments, and other organizations from Beijing Normal University, Tongji University, Beihang University, Shandong University, Ocean University of China, Hohai University, Dalian University of Technology, Ministry of Education of China, China Earthquake Administration, Ministry of Agriculture and Rural Affairs of China, and so on.

The refinement and statistical analysis of the main authors who have carried out international research in dynamic disaster monitoring and early warning are shown in Table 5. As can be seen from Table 5, according to the number of published papers, the top 10 major scholars are Wang, Enyuan (26 articles), Li, Zhonghui (16 articles), Li, Chengwu (9 articles), Li, Xuelong (9 articles), Niu, Yuechuan (8 articles), Lai, Xingping (7 articles), Li, Baolin (7 articles), Cui, Feng (7 articles), He, Xueqiu (6 articles), Liu, Xiao Fei (6 articles).

In addition, Chinese scholars engaged in such research and published more internationally include Qiu Liming, Liu Zhentang, Zhang Jiquan, Xie Beijing, Chen Shaojie, Liu Xianfeng, Dou Linming, Lu Caiping, Chen Nengcheng, Zhang Xin, Shan Pengfei, et al.

### 2.4. The Research Hotspots of China’s International Research on Dynamic Disaster Monitoring and Early Warning

**Figure 2:**
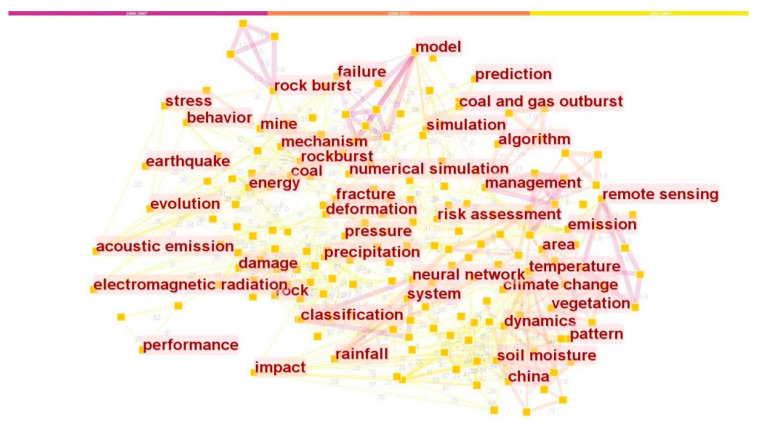
The knowledge map of China’s dynamic disaster monitoring and early warning international research hotspots (2000–2021) (node type).

**Figure 3:**
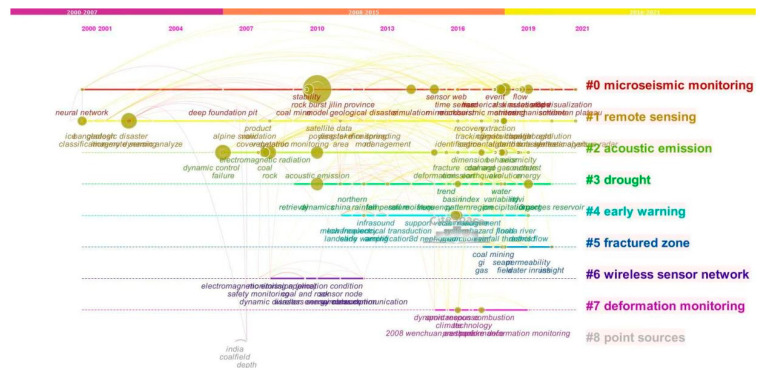
The knowledge map of China’s dynamic disaster monitoring and early warning international research hotspots (2000–2021) (timeline type).

This research focuses on the international research articles published by Chinese scholars within the Web of Science Core Collection database, conducts data analysis and knowledge map visualization analysis of themes and keywords, and explores the hotspots of international research conducted by Chinese scholars in the area of dynamic disaster monitoring and early warning, from 2000 to 2021. Many studies have found that subject headings and keywords are the primary embodiment and summary of the themes and research hotspots [59,60,61,62]. This research uses the CiteSpace software (version 6.1.2 R2) to draw the keywords co-occurrence knowledge maps in related research, which can visually analyze the main node information and network connection layout of the co-occurring hotspots and keywords in related research, and intuitively and deeply understand the hotspots and academic frontiers of related fields [61,62,63]. We take 608 articles by Chinese scholars after searching, downloading, and screening them as basic data, and use CiteSpace software to carry out the visualization analysis of relevant literature. Through visualization analysis, we can gain insight into the information, topics, and keywords of the main nodes, as well as the distribution and classification of the nodes gradually according to the development of the timeline. The drawn knowledge maps are two types of knowledge maps of international research hotspots on dynamic disaster monitoring and early warning in China, node type knowledge maps (Figure 2), and timeline type knowledge maps (Figure 3).

As shown in Figure 2 and Figure 3, the nodes of various networks and sub-networks in the knowledge graph display the topic words and keywords of related research, and the lines existing in the network represent the co-occurrence and co-citation relationship between nodes. Sub-networks within the network also form clusters of corresponding small research areas. From Figure 2 and combined with data analysis, it can be seen that the nodes with high citation frequency are “model” (49 times), “remote sensing” (45 times), “dynamics” (40 times), “rock” (38 times), “failure” (37 times), “prediction” (36 times), “simulation” (35 times), “rock burst” (35 times), “acoustic emission” (32 times), “electromagnetic radiation” (31 times), “coal” (30 times), “China” (29 times), “mine” (28 times), etc. The combination of Figure 3 and software analysis shows that, according to the development of the timeline, except for common research hotspots and keywords, after entering the stage of rapid development in 2018, “system” (25 times), “algorithm” (23 times), and “evolution” (22 times) and other main node themes and keywords indicating systematic and algorithmic comprehensive research, evolution research, and interdisciplinary research begin to appear gradually. On the whole, in these three development stages and each period, there are large networks and sub-networks in relevant fields and sub-fields, as well as large and small themes, research hotspots, and keywords. After clustering analysis of these main nodes and keywords, as shown in the right part of Figure 3, different clusters and small research fields of about nine topics are formed, such as clusters “#0 microseismic monitoring”, “#1 remote sensing”, “#2 acoustic emission”, “#3 drought”, “4# early warning”, “#5 fractured zone”, “#6 wireless sensor network”, “#7 deformation monitoring”, etc.

Through data analysis and visualization analysis of themes and keywords that appeared in the years 2000–2021, this study can explore the international research hotspots of Chinese scholars in dynamic disaster monitoring and early warning. In the initial development period, there are few studies with themes and keywords such as “disaster”, “flood monitoring”, “dynamic”, “coal”, “rock”, etc. Then, in the rising development period, there are some studies with characteristics and scale on themes focusing on “China”, “stability”, and “safety monitoring”. Then, in the rapid development period, there is focus on systematic and comprehensive research, evolutionary research, and interdisciplinary research. On the one hand, Chinese scholars in this field are conducting scientific frontier exploration and sound development, and on the other hand, they have an interactive relationship with the change of disaster events at home and abroad and the layout and promotion of national policies.

## 3. Quantitative Analysis of China’s Dynamic Disaster Monitoring and Early Warning National-Level Policy Documents

### 3.1. The Stage Division of China’s Dynamic Disaster Monitoring and Early Warning National-Level Policy

The quantitative research of policy documents is to transplant the theory and method contents of bibliometrics and scientometrics into the field of policy research, which can analyze the evolution of policy themes, institutional cooperation networks, and intergovernmental relations in terms of structural elements (issuing agencies) and thematic characteristics (policy themes) through policy text data and government website information resources [54,55,56,64]. Quantitative analysis of policy documents can include quantitative analysis and policy analysis of policy theme, policy layout, and policy guidance promotion through the mining of policy keyword information and the drawing of policy keyword co-occurrence maps [65,66,67].

**Figure 4:**
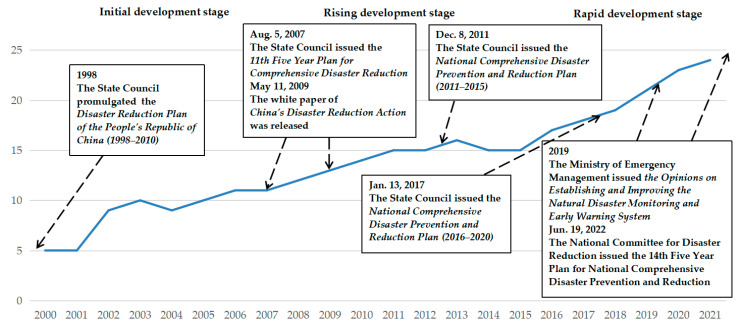
The development trend and stage division of China’s dynamic disaster monitoring and early warning national-level policy (2000–2021).

This study first used Python software (version 3.10) to retrieve, obtain, and download the policies (2000–2021) related to the topic “dynamic disaster monitoring and early warning” from relevant websites such as the State Council of the People’s Republic of China (https://www.gov.cn/, accessed on 10 October 2022) and the Ministry of Emergency Management of the People’s Republic of China (https://www.mem.gov.cn/, accessed on 10 October 2022). In addition, according to the related topics, we searched and selected the policy documents on the website of the “IPOLICY” policy analysis system (http://39.105.58.246/ipolicy/, accessed on 10 October 2022) of the Center for Science, Technology & Education Policy, Tsinghua University. After the retrieval, downloading, cleaning, and filtering of policy document data on relevant topics, 187 national-level theme policy documents in relevant fields were finally obtained. We sorted out and analyzed such policy documents by year and stage, and drew the development trend and stage division diagram of national-level policy documents, as shown in Figure 4.

With the data collection and analysis of 187 national-level policy documents and the stage division analysis related to the research on dynamic disaster monitoring and early warning, in order to better carry out a comparative analysis, this study also divides the development stages of national-level policies in this field into three major stages. Referring to Figure 4, the specific developmental stages and landmark policies are as follows: (1) Initial development stage (2000–2007). At this stage, the Disaster Reduction Plan of the People’s Republic of China (1998–2010) promulgated by the State Council in 1998 is in the implementation period. On 5 August 2007, the State Council issued the 11th Five-Year Plan for Comprehensive Disaster Reduction. However, generally speaking, there are fewer relevant national-level policies during this period, but the overall trend is gradually increasing. (2) Rising development stage (2008–2015). At this stage, the number and speed of overall policies have been steadily improved. On 11 May 2009, the white paper on China’s Disaster Reduction Action was released. Then, on 8 December 2011, the State Council issued the National Comprehensive Disaster Prevention and Reduction Plan (2011–2015). (3) Rapid development stage (2016–2021). At this stage, the overall policy was introduced with great intensity and density, and the policy layout and promotion of most related topics and fields entered a new period. On 13 January 2017, the State Council issued the National Comprehensive Disaster Prevention and Reduction Plan (2016–2020). In 2019, the Ministry of Emergency Management issued the Opinions on Establishing and Improving the Natural Disaster Monitoring and Early Warning System. Furthermore, after the rapid development stage, on 19 June 2022, the National Committee for Disaster Reduction issued the 14th Five-Year Plan for National Comprehensive Disaster Prevention and Reduction. China has been continuously and steadily carrying out the layout and promotion of policies in related fields with the assistance of five-year plans and various supporting policies.

### 3.2. The Quantitative Analysis of China’s Dynamic Disaster Monitoring and Early Warning National-Level Policy Documents and Comparative Study between Policy Keywords and Research Hotspots

**Figure 5:**
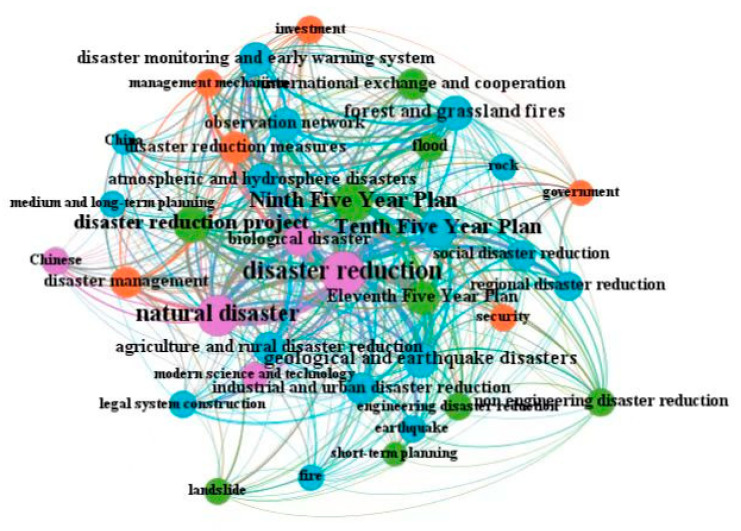
The keyword co-occurrence map of China’s dynamic disaster monitoring and early warning national policy in the initial development stage (2000–2007).

**Figure 6:**
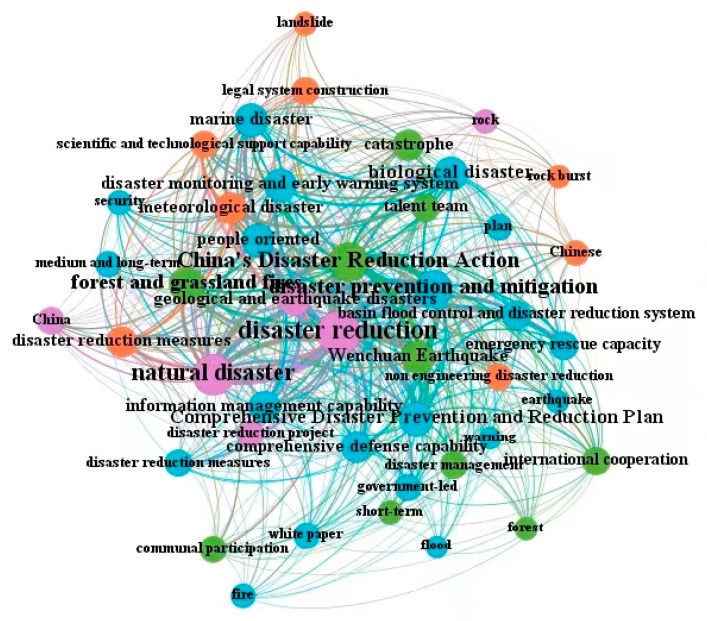
The keyword co-occurrence map of China’s dynamic disaster monitoring and early warning national policy in the rising development stage (2008–2015).

**Figure 7:**
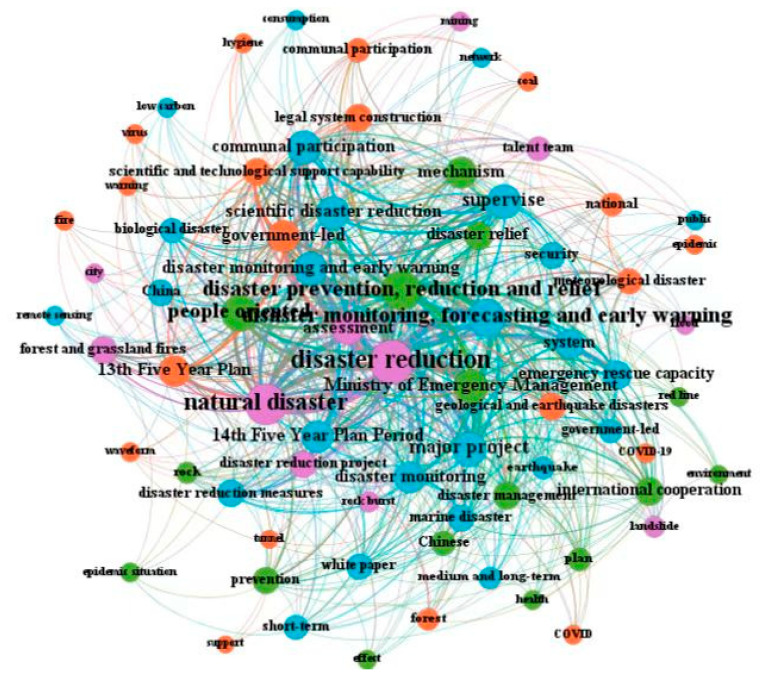
The keyword co-occurrence map of China’s dynamic disaster monitoring and early warning national policy in the rapid development stage (2016–2021).

The quantitative analysis of policy documents is based on a certain amount of policy documents, using the methods and principles of bibliometrics, scientometrics, and visualization analysis to realize the mining of policy topics and the drawing of graph networks for related keywords [64,65,66,67]. Among the theories and methods of this kind of research, the co-occurrence of topics and keywords is one of the critical representation methods of the visual network graph. When two words appear N times in a policy document simultaneously, it is recorded as the N times word co-occurrence, and the number of times indicates the intensity of relevance between two words [65,66,67]. This article uses the co-occurrence method of subject words and keywords to carry out a policy analysis of China’s dynamic disaster monitoring and early warning national-level policy documents, and explore the theme evolution and development process of related policies in this field. According to the three-stage division of the initial development stage, the rising development stage, and the rapid development stage, we have drawn the co-occurrence maps of the three stages of policy keywords (as shown in Figure 5, Figure 6 and Figure 7), and carried out a comparative analysis based on the international research hotspots of Chinese scholars in this field.

Figure 5 shows that in the initial development stage of dynamic disaster monitoring and early warning policy (2000–2007), the relevant national policies in this period were mainly macro policies. In addition to the conventional “disaster reduction”, “natural disaster”, “dynamic disaster monitoring and early warning”, the core theme words were mainly the “Ninth Five Year Plan”, “Tenth Five Year Plan”, and other relevant national disaster reduction plans implemented and promulgated in succession during this period. Some related policy themes were “engineering disaster reduction” and “non-engineering disaster reduction”, “disaster reduction project”, and “disaster reduction measures” which were the main types of disaster reduction work, and some themes were “atmospheric and hydrosphere disasters”, “geological and earthquake disasters”, and “biological disaster” which were the main types of disaster. There were also references to disaster reduction at the social or regional level, such as the keywords “agriculture and rural disaster reduction”, “industrial and urban disaster reduction”, “regional disaster reduction”, and “social disaster reduction”. Meanwhile, the international research of Chinese scholars in this period was also in the initial stage, and the research hotspots and keywords mostly focused on the specific theories and methods of disaster reduction and early warning research in the research field of dynamic disaster monitoring and early warning, such as “flood monitoring”, “remote sensing”, “dynamic analysis”, “GIS”, “neural network”, “monitoring and assessment”, “model”, “inversion analysis”, and “safety degree”. It could be seen that during this period, there were not many policies issued at the national level, which tended to be macro-oriented. Although the international research of Chinese scholars was still in the initial stage of development, the number was not significant, but the research was relatively focused. During this period, the policy theme in this field deviated from the research hotspots in the academic circles, which belonged to the relationship between top-down design and concrete implementation of research work. The policy themes and academic research during this period all reflected the emphasis on and development of international cooperation and exchanges.

As shown in Figure 6, during the rising development stage (2008–2015) of dynamic disaster monitoring and early warning policy, words such as “disaster reduction”, “disaster prevention and mitigation”, “natural disaster”, “China’s Disaster Reduction Action”, “Wenchuan Earthquake”, “people oriented”, “disaster monitoring and early warning system” are the core keywords of this stage. After the Wenchuan earthquake in Sichuan on 12 May 2008, the national policy has paid more and more attention to the prevention, monitoring, and early warning of earthquakes, geological disasters, and natural disasters. In 2009, the Chinese government decided to designate May 12 every year as the national “Disaster Prevention and Mitigation Day” and released China’s Disaster Reduction Action White Paper, emphasizing that the Chinese government is “people-oriented” and always puts the protection of public life and property safety first, and integrates disaster reduction into economic and social development plans as an important guarantee for achieving sustainable development. In addition to the re-emphasis on various types of natural disasters (“meteorological disaster”, “geological and earthquake disasters”, “biological disaster”, “forest and grassland fires”, “marine disaster”, etc.), the policy keywords in this period emphasize the need to strengthen the capacity building of disaster reduction and prevention, the construction of disaster early warning monitoring systems, the construction of emergency management systems, legal system institutional mechanism construction, such as the emergence and increase in the policy keywords “disaster monitoring and early warning system”, “information management capability”, “comprehensive defense capability”, “emergency response capacity”, “basin flood control and disaster reduction system”, “legal system construction”, and “disaster management”.

At this stage, the international research of Chinese scholars was also gradually enriched, and there were interactions with policies at all levels and in all fields. For example, some scholars conducted research on postdisaster assessment, secondary injury analysis, and prevention of the Wenchuan earthquake, and gave data analysis and policy recommendations, forming a good interaction with the introduction, layout, and implementation of policies before and after the Wenchuan earthquake [34,35]. The coincidence and interaction between policy themes and academic research hotspots began to increase, the relationship between political initiatives and academic research began to become close, and the trend of international cooperation was also strengthening. On 8 December 2011, the State Council issued the “National Comprehensive Disaster Prevention and Mitigation Plan (2011–2015)”, which indicated that in the later stage of this period, the state summed up the achievements and experiences of the 11th Five-Year Plan period and began to lay out the layout for the development of the 12th Five-Year Plan period. The plan specifically emphasized that disaster prevention and mitigation should be based on the overall situation of national economic and social development, make overall plans for the development of comprehensive disaster prevention and mitigation, accelerate the capacity building, and constantly improve the comprehensive disaster prevention and mitigation system. The issuance and implementation of policies were closely related to the early warning and prevention of natural disasters, disaster reduction, prevention work, system construction, scholars’ research work, and policy recommendations at this stage.

As shown in Figure 7, in the rapid development stage (2016–2021) of dynamic disaster monitoring and early warning policy, keywords such as “disaster reduction”, “disaster prevention, reduction and relief”, “natural disaster”, “disaster monitoring, forecasting and early warning”, “Ministry of Emergency Management”, “disaster monitoring and early warning”, “people-oriented”, “major project” are the core keywords of this stage. It should be noted that the specific references in many policy documents have changed from the original “disaster prevention and reduction”, “disaster prevention and mitigation” to “disaster prevention, reduction and relief”, especially the addition of “disaster relief”. There are also many policy themes that not only emphasize “disaster monitoring and early warning” but also “disaster monitoring, forecasting and early warning”. The richness and change of these policy themes are also reflected in many scholars’ policy analysis articles and review articles. On 13 January 2017, the State Council issued the “National Comprehensive Disaster Prevention and Mitigation Plan (2016–2020)”, which indicated that at the beginning of this stage, the state summed up the achievements and experiences in the 12th Five-Year Plan period and began to lay out the layout for the development in the 13th Five-Year Plan period. In the plan, it was specially emphasized that prevention should be the focus and prevention should be combined with rescue. It was also emphasized that normal disaster reduction and abnormal disaster relief should be unified. Efforts should be made to realize the transformation from focusing on postdisaster relief to focusing on predisaster prevention, from dealing with a single disaster to comprehensive disaster reduction, and from reducing disaster losses to reducing disaster risks. Efforts should be made to build disaster prevention, mitigation, and relief systems and mechanisms that are compatible with the new stage of economic and social development, and to comprehensively improve the comprehensive prevention capability of the whole society against natural disasters.

In March 2018, the Ministry of Emergency Management of the People’s Republic of China was established. In 2019, the Ministry of Emergency Management issued the Opinions on Establishing and Improving the Natural Disaster Monitoring and Early Warning System. This document requires in-depth implementation from the national level to local provinces and cities and establishes a nationwide systematic monitoring and early warning system for natural disasters. It is a landmark policy on dynamic disaster monitoring and early warning in China at this stage, which has made a top-down design for China’s monitoring, early warning, forecasting, and prevention of natural disasters. The relevant policy keywords “Ministry of Emergency Management” and “disaster monitoring and early warning system” also appeared in many scholars’ research articles and review articles. Overall, in the rapid development stage of dynamic disaster monitoring and early warning policy, the relationship between academic research and policy top-level design has achieved rich and benign interaction, and the coincidence degree between scholars’ research hotspots and policy themes is becoming higher and higher. While conducting scientific research, experts and scholars are more involved in the government’s policy formulation, consultation, and suggested activities. At this stage, the policy theme has an unprecedented interactive relationship with scholars’ research hotspots, realizing a great degree of coincidence and linkage.

In the past two or three years, including the year 2022, Chinese scholars’ international research on dynamic disaster monitoring and early warning has also begun to show more research on carbon neutrality and the COVID-19 epidemic, which involves the fields of emergency management, environmental research, and public health research, and is also the content of disaster prevention and mitigation and emergency management that China’s policy has focused on in recent years [6,20,31,42,46,58]. It is found that in recent years, the relevant policies and research contents of dynamic disaster monitoring and early warning are constantly enriched, and the scope is constantly expanding, and the relationship between academic research and policy themes is becoming closer and closer.

## 4. Discussion

This article discusses the interaction between academia and politics through the comparative analysis of international research hotspots and policy keywords of Chinese scholars in dynamic disaster monitoring and early warning. The “international” research mentioned in this study refers to the results of international cooperation papers of Chinese scholars mainly published in international journals and the core collection of the Web of Science database. In the second section, we conduct a preliminary overview of relevant research from the macro and meso levels, and carry out quantitative analysis and knowledge map visualization analysis. Then, in the third section, we carry out a quantitative analysis of policy literature, and a comparative study of policy and research keywords. As a matter of fact, Chinese scholars also have some valuable articles published in domestic journals, and this study also uses and refers to some Chinese articles [3,4,6]. Many high-yield authors and major core institutions in China mentioned in this study have many articles in Chinese and English, and their articles have certain integration and overlap in research topics, research projects, and innovation transformation. At present, many domestic journals have been internationalized one after another, and similar English versions have been published. In contrast, the English articles of Chinese scholars are better representative in terms of quality and quantity. In order not to affect the mixture and expression of Chinese and English articles in general, this study mainly analyzes international articles. In general, this research will not affect the expression of the conclusion of this paper. In recent years, some scholars have successively carried out comparative analysis on international research and domestic policies in other fields, and the structure and conclusions of these studies are also exploratory and reasonable [68,69].

Through the knowledge mapping analysis of academic research literature, and the quantitative analysis of the latest policy literature, combined with recent academic research and policy trends, this study suggests that the focus and direction of Chinese academic research and policy in recent years lie in the prevention and control of COVID-19, climate change and carbon neutrality, dynamic disaster early warning, emergency response and emergency management, national security, and the continuous promotion of the construction of a community with a shared future for mankind.

## 5. Conclusions

In the first part of the research, 608 Chinese scholars’ articles on dynamic disaster monitoring and early warning downloaded from the database were analyzed mathematically and visually using the methods of scientometrics and knowledge map visualization analysis. We sorted out the articles of international research by Chinese scholars in this field annually and divided them into stages, and we selected six highly cited papers co-published by Li Xuelong, Chen Shaojie, and other scholars. Then, we sorted out and analyzed the main discipline categories, countries and regions, main institutions, and scholars of China’s international research on disaster dynamic monitoring and early warning. In the visual analysis, we used CiteSpace software to draw and analyze the knowledge maps of research related to disaster dynamic monitoring and early warning, and explored the hotspot information and evolution process of international research of Chinese scholars at different development stages.

In the second part of the research, we screened and sorted out the policy documents retrieved and downloaded from the websites of relevant government departments and the relevant government document databases of the Center for Science, Technology & Education Policy, Tsinghua University. By using the method of quantitative analysis of policy documents, the stage division of policy document data and the visual analysis of the policy keyword co-occurrence maps were carried out. The evolution stage division of the policy theme is consistent with the stage division of international research hotspots of Chinese scholars, which can be compared and analyzed. The three stages are divided into the initial development stage (2000–2007), the rising development stage (2008–2015), and the rapid development stage (2016–2021). This paper makes a comparative study of the three stages of policy themes and international research hotspots.

This study carried out a comparative study. Through the combination of visual analysis of knowledge maps and quantitative analysis of policy documents, it objectively and accurately developed the similarities and differences and interactions between relevant research hotspots and policy themes, and further in-depth analysis and research were carried out in combination with literature review and policy analysis. For example, after entering the stage of rapid development, except for regular topics and keywords, “system” (25 times), “algorithm” (23 times), and “evolution” (22 times) and other main node themes and keywords indicating systematic and algorithmic comprehensive research, evolution research, and interdisciplinary research begin to appear gradually. The studies in the rapid development stage focus on systematic and comprehensive research, evolutionary research, and interdisciplinary research. Meanwhile, in the rapid development stage (2016–2021) of dynamic disaster monitoring and early warning policy, keywords such as “disaster reduction”, “disaster prevention, reduction and relief”, “natural disaster”, “disaster monitoring, forecasting and early warning”, “Ministry of Emergency Management”, “disaster monitoring and early warning”, “people-oriented”, “major project” are the core keywords of this stage. At this stage, the policy theme has an unprecedented interactive relationship with scholars’ research hotspots, realizing a great degree of coincidence and linkage.

It is found that in the first stage, the initial development stage (2000–2007), the international research literature is smaller and more focused, while the national policy documents are fewer and more macroscopic, so the research hotspots are somewhat alienated from the policy keywords. In the second stage, the rising development stage (2008–2015), after the Wenchuan earthquake, the relationships between research hotspots and policy keywords begins to become closer, mostly concentrated in the fields of geology, engineering disasters, meteorological disasters, natural disasters, etc. Meanwhile, research hotspots also focus on cutting-edge technologies and theories, while the national-level policy keywords pay more attention to overall governance and macro promotion, but their coincidence and tightness increase a lot. In the third stage, the rapid development stage (2016–2021), with the state’s continuous emphasis on disaster monitoring and early warning, disaster reduction, disaster prevention and relief work, and continuous policy promotion and top-down design, the government set up the Ministry of Emergency Management and gradually improved the disaster early warning and monitoring system. The research hotspots and policy keywords are mutually integrated with each other, with a high degree of coincidence, thus realizing the organic linkage and mutual promotion between academic research and political deployment. In general, with the continuous improvement of the national disaster early warning and monitoring system, the orderly advancement of the national comprehensive disaster reduction and prevention plan, and the improvement of the emergency management system and supporting policies, laws, and regulations, the international research hotspots of Chinese scholars in this field are increasingly closely linked with national policy themes, forming a mutually reinforcing positive relationship. In recent years, with the emergence and continuation of COVID-19 and the orderly progress of carbon neutrality and carbon peaking, public health events and environmental governance issues have gradually become the research hotspots and policy focus of emergency management and disaster early warning monitoring and prevention.

## Figures and Tables

**Table 1:** The highly cited articles of China’s dynamic disaster monitoring and early warning international research (2000–2021).

Highly Cited Papers	Citations
1. Levin, N.; Kyba, C.C.M.; Zhang, Q.; de Miguel, A.S.; Roman, M.O.; Li, X.; Portnov, B.A.; Molthan, A.L.; Jechow, A.; Miller, S.D.; Wang, Z.; Shrestha, R.M.; Elvidge, C.D. Remote sensing of night lights: A review and an outlook for the future. Remote Sens. Environ. 2020, 237, 111443. [35]	207
2. Li, X.; Chen, S.; Wang, E.; Li, Z. Rockburst mechanism in coal rock with structural surface and the microseismic (MS) and electromagnetic radiation (EMR) response. Eng. Fail. Anal. 2021, 124, 105396. [47]	85
3. Zhang, Y.; Tang, J.; Liao, R.; Zhang, M.; Zhang, Y.; Wang, X.; Su, Z. Application of an enhanced BP neural network model with water cycle algorithm on landslide prediction. Stoch. Env. Res. Risk A. 2021, 35, 1273–1291. [46]	85
4. Li, X.; Chen, S.; Liu, S.; Liu, Z. AE waveform characteristics of rock mass under uniaxial loading based on Hilbert-Huang transform. J. Cent. South Univ. 2021, 28, 1843–1856. [44]	82
5. Li, X.; Chen, S.; Zhang, Q.; Gao, X.; Feng, F. Research on theory, simulation and measurement of stress behavior under regenerated roof condition. Geomech. Eng. 2021, 26, 49–61. [45]	77 (Hot Paper)
6. Fan, J.; Meng, J.; Ludescher, J.; Chen, X.; Ashkenazy, Y.; Kurths, J.; Havlin, S.; Schellnhuber, H.J. Statistical physics approaches to the complex Earth system. Phys. Rep. 2021, 896, 1–84. [31]	31

**Table 2:** The main disciplinary categories of China’s dynamic disaster monitoring and early warning international research (2000–2021).

No.	Web of Science Categories	Paper Numbers
1	Geosciences Multidisciplinary	117
2	Environmental Sciences	105
3	Engineering Electrical Electronic	83
4	Remote Sensing	82
5	Water Resources	51
6	Computer Science Information Systems	49
7	Engineering Civil	47
8	Imaging Science Photographic Technology	46
9	Engineering Geological	43
10	Meteorology Atmospheric Sciences	39

**Table 3:** The main cooperative countries and regions of China’s dynamic disaster monitoring and early warning international research (2000–2021).

No.	Countries/Regions	Paper Numbers
1	USA	39
2	Australia	10
3	Japan	10
4	Pakistan	9
5	England	8
6	Germany	6
7	Canada	5
8	Spain	4
9	Austria	4
10	Belgium	4

**Table 4:** The main institutions of China’s dynamic disaster monitoring and early warning international research (2000–2021).

No.	Institutions (Affiliations)	Paper Numbers
1	China University of Mining Technology	95
2	Chinese Academy of Science	87
3	University of Chinese Academy of Science CAS	30
4	Wuhan University	28
5	Shandong University of Science and Technology	26
6	China University of Geosciences	24
7	Chongqing University	22
8	Helmholtz Association	18
9	University of Science Technology Beijing	17
10	Xi’an University of Science and Technology	17

**Table 5:** The main scholars of China’s dynamic disaster monitoring and early warning international research (2000–2021).

No.	Scholars (Researchers, Authors)	Paper Numbers
1	Wang, Enyuan	26
2	Li, Zhonghui	16
3	Li, Chengwu	9
4	Li, Xuelong	9
5	Niu, Yuechuan	8
6	Lai, Xingping	7
7	Li, Baolin	7
8	Cui, Feng	7
9	He, Xueqiu	6
10	Liu, Xiao Fei	6
